# Potential synergistic antihyperglycemic effects of co-supplemental Amla and Olive extracts in hyperlipidemic adults with prediabetes and type 2 diabetes: results from a real-life clinical study

**DOI:** 10.3389/fnut.2024.1462292

**Published:** 2024-10-03

**Authors:** Hermans Michel P., Sylvie A. Ahn, Michel F. Rousseau, Laurence Seidel, Adelin Albert, Isabelle Janssens, Yvan Dierckxsens, Amjad Khan

**Affiliations:** ^1^Division of Endocrinology and Nutrition, Cliniques Universitaires St-Luc and Institut de Recherche Expérimentale et Clinique (IREC), Université Catholique de Louvain, Brussels, Belgium; ^2^Division of Cardiology, Cliniques Universitaires St-Luc and Pôle de Recherche Cardiovasculaire, Institut de Recherche Expérimentale et Clinique (IREC), Université Catholique de Louvain, Brussels, Belgium; ^3^B-STAT, Centre Hospitalier Universitaire, Liège, Belgium; ^4^Laboratoire Tilman, Baillonville, Belgium; ^5^Department of Oncology, University of Oxford, Oxford, United Kingdom; ^6^Department of Biochemistry, Liaquat University of Medical and Health Sciences (LUMHS), Jamshoro, Pakistan

**Keywords:** Cholesfytol NG^®^, Amla, Olive, antihyperglycemic, antihyperlipidemic

## Abstract

**Background:**

Hyperglycemia and type 2 diabetes mellitus (T2DM) pose a significant risk for cardiovascular diseases and associated complications in individuals with hyperlipidemia. Statin therapy, effective in reducing cholesterol and cardiovascular risks, paradoxically increases incident T2DM risk due to its adverse impact on glucose homeostasis. Therefore, there is a pressing need for safe, and effective adjunctive or alternative therapies to manage hyperglycemia in hyperlipidemic individuals. There is growing body of pharmacological evidence suggesting that Amla and Olive extract supplementation can be beneficial in managing hyperglycemia in individuals with hyperlipidemia.

**Objective:**

The present study aimed to assess for the first time the potential synergistic antihyperglycemic effects of a daily co-supplementation of 1,000 mg Amla fruit and 50 mg Olive fruit standardized extracts (Cholesfytol NG^®^) over a 2-months period in hyperlipidemic adults with T2DM or prediabetes.

**Methods:**

This retrospective cross-sectional observational study analyzed treatment outcomes in 191 hyperlipidemic adults under the care of their physicians at 57 General Practitioner clinics in Belgium during real-life clinical practice between March 19, 2020, and January 31, 2022. These participants received Cholesfytol NG^®^ as supplementary therapy to improve their metabolic health. The supplement was prescribed in an open-label, non-randomized manner, tailored to each participant’s need.

**Results:**

After 2-months of Cholesfytol NG^®^ supplementation, participants showed significant reductions in glycemia levels: in the T2DM group, levels decreased by 42.7 ± 17.9 mg/dL (27.9%, *p* < 0.0001), and in the prediabetic group, by 2.26 ± 11.5 mg/dL (4.7%, *p* = 0.0020). Conversely, no significant change was observed in participants with normal baseline glycemia (1.55 ± 10.3 mg/dL, *p* = 0.088). Overall, glycemia levels decreased from 96.4 ± 18.2 mg/dL to 94.0 ± 13.5 mg/dL (mean decrease of 2.4 ± 14.5 mg/dL, *p* < 0.0001). The supplement was well tolerated and no side-effects, serious adverse events, or treatment-emergent effects were reported.

**Conclusion:**

The findings of this real-life clinical study highlight the potential synergistic antihyperglycemic effects of co-supplementation with Amla and Olive fruit extracts in managing hyperglycemia, particularly in individuals with hyperlipidemia. These results suggest that this botanical combination may help mitigate risks associated with hyperglycemia and cardiovascular disease in hyperlipidemic population.

**Clinical trial registration:**

ClinicalTrials.gov, NCT06187298.

## 1 Introduction

Hyperglycemia and type 2 diabetes mellitus (T2DM) are significant risk for cardiovascular diseases (CVD) in individuals with hypercholesterolemia, potentially leading to coronary artery disease, stroke, and other complications. Although statin therapy lowers low-density lipoprotein cholesterol (LDL-C) and reduces CVD risk, it is associated with an increased risk of incident prediabetes and T2DM (1–3). Elevated blood glucose levels in hyperlipidemic individuals are linked to insulin resistance, oxidative stress, subclinical inflammation, and lipotoxicity, which heighten the risk of T2DM and its complications, including cardiovascular outcomes, neuropathy, nephropathy, and retinopathy.

The concurrent use of statin therapy, which can cause statin-associated muscle symptoms (SAMS) and potential liver damage, with glucose-lowering medications, which may induce gastrointestinal disturbances, presents challenges in managing multimorbidity involving hyperlipidemia and hyperglycemia. These side effects can exacerbate each other, leading to decreased patient adherence and worsening overall health outcomes, including increased risk of cardiovascular events and reduced quality of life. Given these challenges, there is an pressing need for safe and effective adjunctive or alternative therapies to improve management and patient outcomes.

**Figure 1:**
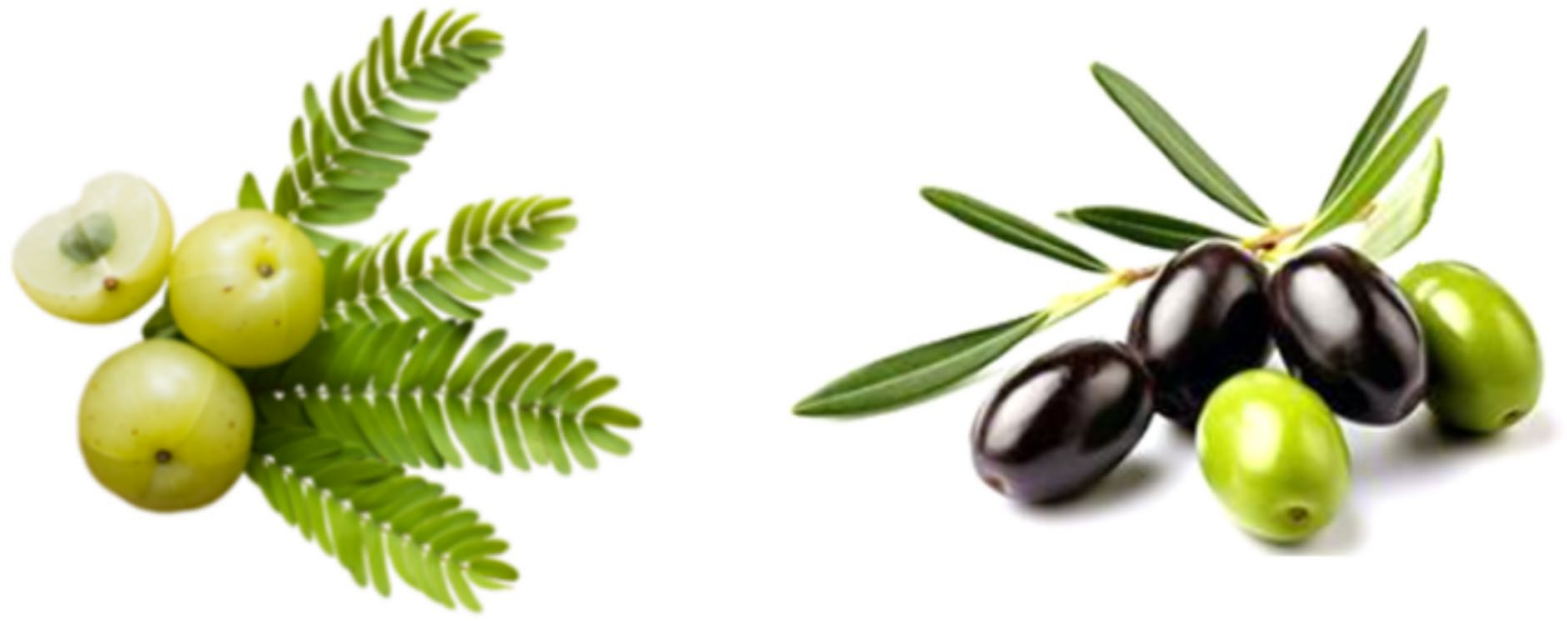
The potential synergistic antihyperglycemic pharmacological effects of co-supplementation of standardized extracts of Amla (*Emblica officinalis*, 1,000 mg) (left) and Olive (*Olea europaea*, 50 mg) (right) fruits (Cholesfytol NG^®^) for 2-months in hyperlipidemic adults was explored in the present clinical study.

Nutraceuticals have gained attention as potential adjuncts to conventional therapies due to their natural origin and favorable safety profiles. Among these (4), Amla (*Emblica officinalis*) (5), commonly known as Indian gooseberry, and Olive (*Olea europaea*) (Figure 1) extracts have shown promising potential antihyperglycemic effects in recent studies (6–8). Amla is rich in vitamin C and polyphenols (including tannins, flavonoids, gallic acid, ellagic acid, and emblicanin A and B) (9–11), and is known for its antioxidant, antihyperglycemic, and cholesterol-lowering benefits (12). The potential antihyperglycemic effects of Amla are believed to be due to its high polyphenols contents, particularly tannins and flavonoids (4, 12–16). Its potential antihyperglycemic pharmacological effects likely involve multiple mechanisms, including modulation of glucose homeostasis, inhibition of carbohydrate-metabolizing enzymes, and antioxidant properties (4, 12–17). Olive extracts, containing phenolic compounds such as oleuropein, hydroxytyrosol, and tyrosol, contribute to antihyperglycemic, antioxidant and anti-inflmmatory effects by enhancing insulin sensitivity, modulating glucose metabolism, and inhibiting carbohydrate-hydrolyzing enzymes (18–20).

The supplemetal combination of Amla and Olive extracts may provide synergistic benefits for blood glucose control, particularly in hypercholesterolemic patients, by addressing both glucose and lipid metabolism comprehensively. This retrospective cross sectional observational study conducted under real-life clinical settings in Belgian General Practitioner (GP) clinics, aims to assess the potential synergistic antihyperglycemic effects of combined supplemental Amla and Olive extracts (Cholesfytol NG®) in hyperlipidemic adults including prediabetes or T2DM. Previous research reported significant antihyperlipidemic effects of this nutritional combination (21). This study extends those findings by evaluating its impact on glycemic control, offering evidence for integrating this nutritional combination into therapeutic regimens as a safe and effective strategy for improving metabolic health and reducing the risk of T2DM and cardiovascular diseases.

## 2 Materials and methods

This retrospective cross-sectional observational study examined the treatment outcomes of a cohort of hypercholesterolemic adults seen by their physicians in real-life clinical practice at 57 GP clinics in Belgium from March 19, 2020, to January 31, 2022. No control group was used. Participants received oral Cholesfytol NG® (Tilman s.a., Baillonville, Belgium) supplemental therapy as part of routine care to improve their metabolic health. Each Cholesfytol NG® tablet contains 500 mg of Amla fruit extract standardized to ≥60.0% hydrolyzable tannins (including emblicanin A and B, punigluconin, and pedunculagin) and 25 mg of Olive fruit extract standardized to 20% hydroxytyrosol (5 mg), among other ingredients. The recommended dosage was two tablets daily (1,000 mg Amla and 50 mg Olive fruit extracts) for 2 months.

The supplement was prescribed to participants in an open-label, non-randomized manner, tailored to each individual’s needs. No specific instructions were given to participants to modify their routine diet during the 2-month supplement therapy period. Administration followed Good Clinical Practice (GCP) guidelines. Since the supplement was already available in Belgium, its use did not alter the participants’ standard of care or require additional diagnostic or follow-up procedures. Participants’ demographic and clinical data were recorded in a case report form. Participants’ blood glucose levels were measured using blood glucose meters at their GP clinics, both at baseline (T_0_) and at the 2-month follow-up appointment (T_1_).

**Figure 2:**
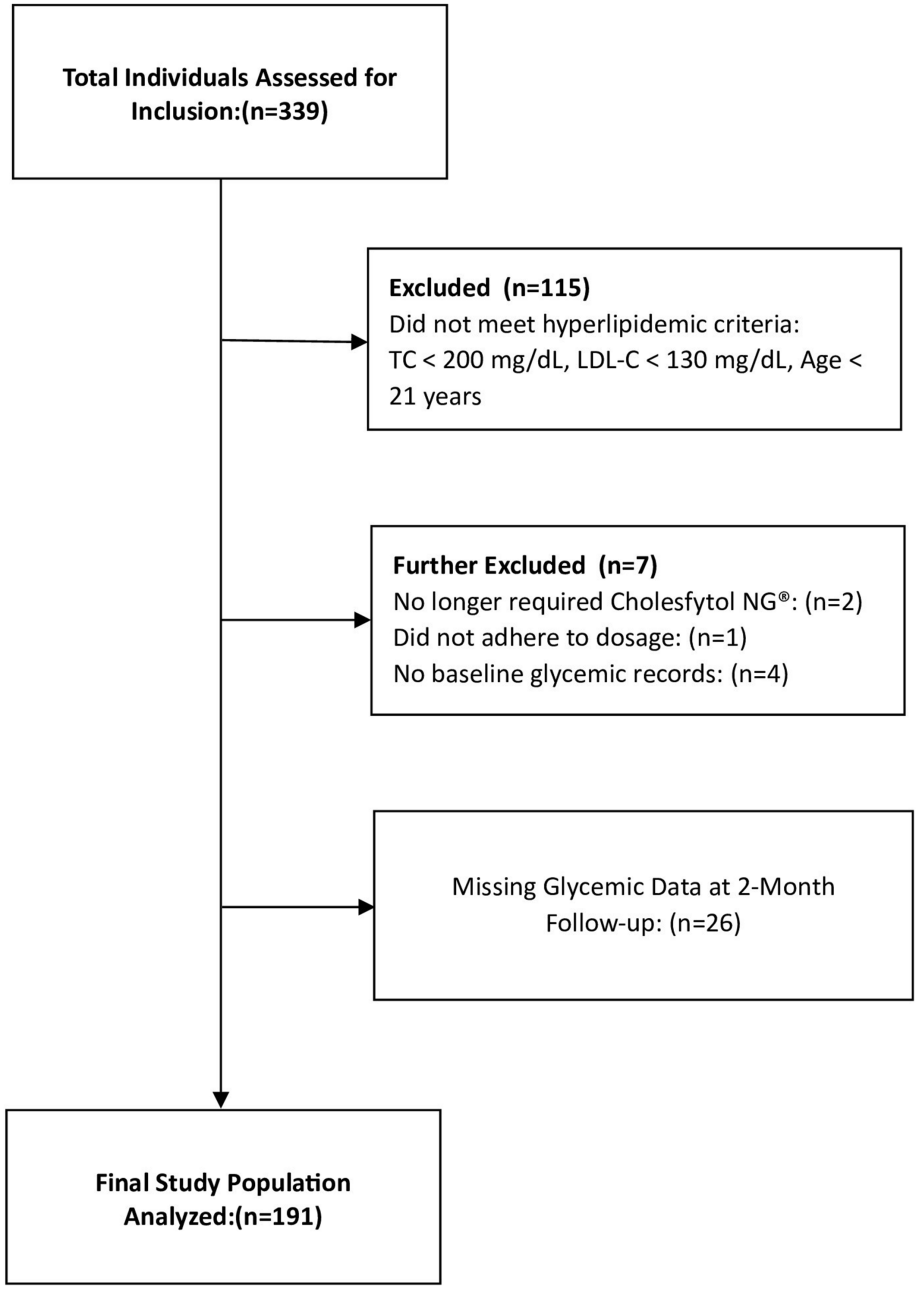
Study flowchart.

To evaluate the potential therapeutic effects of Cholesfytol NG® on glycemic control in hypercholesterolemic individuals, our analysis of the treatment outcome focused on those who met the following criteria: (1) age ≥ 21 years; (2) both male and female; (3) a diagnosis of hypercholesterolemia (total cholesterol [TC] ≥ 200 mg/dL and low-density lipoprotein cholesterol [LDL-C] ≥ 130 mg/dL); and (4) receipt of Cholesfytol NG® at a dosage of two tablets per day for 2 months. According to Belgian regulations, institutional review board (IRB) ethics approval was not required for this retrospective cross sectional observational study, which involved the analysis of existing patients data (22). The study was registered at ClinicalTrials.gov (ID NCT06187298). The study analysis included a total of 191 participants, as outlined in the study flowchart (Figure 2).

## 3 Statistical analysis

Results are expressed as mean and standard deviation (SD). Glycemia mean levels before and after treatment were compared by paired Student t-test. Differences between means were considered significant at the 5% level (*p* < 0.05). Calculations were done with SAS version 9.4 software.

## 4 Results

Participants mean age was 61.4 ± 12.4 years, of whom 37.9% were men. Based on baseline glycemic levels (Table 1), there were 9 (4.7%) participants with T2DM (≥ 126 mg/dL), 51 (26.7%) participants at risk of developing T2DM (prediabetes, 100–125 mg/dL), and 131 (68.6%) participants not at risk (< 100 mg/dL, normal levels).

**Table 1:** Evolution of the participants glycemia levels between baseline (T_0_) and after 2-month Cholesfytol NG^®^ supplementation (T_1_) (*N* = 191).

		T_0_ glycemia	T_1_ glycemia	Glycemia absolute change	
		(mg/dL)	(mg/dL)	(mg/dL)	
Glycemia levels (mg/dL)	*N*	Mean ± SD	Mean ± SD	Mean ± SD	*p*-value
≥ 126 (T2DM)	9	154 ± 25.8	111 ± 32.9	−42.7 ± 17.9	<0.0001
100–125 (prediabetic)	51	107 ± 6.69	102 ± 11.8	−2.26 ± 11.5	0.0020
<100 (normal glycemia)	131	88.3 ± 9.12	89.8 ± 9.24	1.55 ± 10.3	0.088
Overall cohort	191	96.4 ± 18.2	94.0 ± 13.5	−2.35 ± 14.5	0.026

Table 1 outlines the glycemic outcomes of the supplemental therapy. After 2-month Cholesfytol NG® supplementation, in the T2DM group, glycemia levels decreased significantly by 42.7 ± 17.9 mg/dL, corresponding to a substantial 27.9% improvement (*p* < 0.0001). Similarly, in the prediabetes group, glycemia levels dropped by 2.26 ± 11.5 mg/dL, or 4.7% (*p* = 0.0020). In contrast, no significant change was noted for participants with baseline glycemia level < 100 mg/dL (normal glycemia) (1.55 ± 10.3 mg/dL, *p* = 0.088), indicating that the supplement’s effect may be more relevant in participant’s population with elevated baseline glycemia levels. Overall, glycemia levels decreased significantly from 96.4 ± 18.2 mg/dL at baseline to 94.0 ± 13.5 mg/dL after the 2-month treatment, with a mean decrease of 2.4 ± 14.5 mg/dL (*p* < 0.0001), corresponding to a 2.5% clinical improvement. Importantly, none of the participants reported any side effects, treatment-emergent effects, or serious adverse events during or at the 2-month follow-up period, indicating a favorable safety and tolerability profile for the supplement.

## 5 Discussion

This retrospective cross sectional observational study, conducted under real-life clinical settings provides valuable insights into the potential antihyperglycemic effects of supplemental combined Amla and Olive fruit extracts in hypercholesterolemic healthy population, including those with prediabetes and T2DM. Over the 2-month supplementation period with Cholesfytol NG®, we observed clinically significant glycemic improvements, particularly in participants with elevated baseline glucose levels. In the T2DM group, glycemia decreased by 42.7 ± 17.9 mg/dL, a 27.9% reduction (*p* < 0.0001), while the prediabetes group saw a reduction of 2.26 ± 11.5 mg/dL or 4.7% (*p* = 0.0020). Importantly, no significant glycemic change was noted in normoglycemic individuals, indicating that the supplement’s effect may be specific to those with dysregulated glucose metabolism. The supplement’s favorable safety profile further enhances its clinical relevance, particularly for hypercholesterolemic individuals at increased risk for T2DM and cardiovascular diseases. The observed improvements in glycemia suggest that the Amla and Olive supplement combination could be an effective adjunctive therapy for managing blood glucose levels and may help (contribute) in mitigating the risk of statin-induced incident hyperglycemia/T2DM, all while being well-tolerated and safe (21).

The observed glycemic control may be attributed to the synergistic mechanisms of the diverse bioactive constituents in both Amla and Olive extracts when combined. Amla, rich in hydrolyzable tannins such as emblicanin-A, emblicanin-B, punigluconin, and pedunculagin (9–11)—types of polyphenols—contributes to improved insulin sensitivity, regulation of glucose homeostasis, and reduction of oxidative stress (4, 12–16). The polyphenols in Amla, particularly tannins and flavonoids, are believed to help control glucose homeostasis through several suggested mechanisms. These include antioxidant properties, inhibition of carbohydrate-metabolizing enzymes, suppression of the polyol pathway, reduction of advanced glycation end product formation, mitigation of mitochondrial dysfunction, promotion of *β*-cell function and number, and modulation of poly (ADP-ribose) polymerase (PARP) and poly (ADP-ribose) glycohydrolase (PARG) activation (9–11, 17).

Olive extracts, on the other hand, are rich in phenolic compounds, particularly oleuropein and hydroxytyrosol (6). The potential antihyperglycemic effects of these Olive-derived phenolics are believed to be mediated through several suggested mechanisms. These include enhancing glucose uptake in peripheral tissues, inhibiting carbohydrate-hydrolyzing enzymes such as *α*-amylase and α-glucosidase, and modulating insulin-related signaling pathways (6, 18, 23).

When combined, the bioactive constituents from both Amla and Olive extracts likely work synergistically (24–26), leading to a more potent antihyperglycemic effect than either extract alone. This combination potentially offers a comprehensive approach to managing hyperglycemia, leveraging the complementary and multidirectional actions of both extracts. This dual-action strategy may provide enhanced therapeutic benefits for individuals with hypercholesterolemia and those at risk of developing T2DM.

The results of the present study align with findings previously reported for individual Amla and Olive extracts supplementation. Several clinical trials have highlighted the potential of Amla fruit extract in managing T2DM and improving lipid profiles (8, 27–34). For instance, newly diagnosed T2DM patients taking 1 or 2 g of aqueous Amla fruit concentrate daily for 3 months showed significant reductions in fasting blood glucose (FBG), postprandial blood glucose (PPBG), and hemoglobin A1c (HbA1c) levels, with effects comparable to metformin (31). Similarly, Amla fruit powder at doses of 1, 2, or 3 g per day for 3 weeks reduced FBG and PPBG, with effects similar to glibenclamide in healthy adults with T2DM (27). Additionally, Amla fruit extract at 1 g per day for 3 months alleviated endothelial dysfunction by reducing oxidative stress markers, with effects akin to atorvastatin in T2DM patients (33). Amla extract at 500 mg per day twice for 4 months also significantly improved lipid profiles in hypercholesterolemic individuals (28). Other studies using high doses of Amla in fruit powder, fresh fruit, and juice form have similarly reported significantly improved glycemic and lipid profiles, comparable to metformin, glimepiride, and glibenclamide (29, 30, 32, 34). The observed antihyperglycemic and antihyperlipidemic effects of Amla are further supported by extensive animal model research (15). Overall, there is substantial evidence suggesting that Amla fruit extract is a promising natural pharmacotherapy for the prevention and management of hyperglycemia and T2DM.

Several clinical trials have also investigated the effects of various Olive plant products on glucose metabolism and related parameters (7, 8, 35). These studies have demonstrated that Olive polyphenols can improve blood glucose levels in prediabetes, reduce markers of lipid peroxidation, and lower blood pressure in hypertensive individuals (8). For instance, supplementation with Olive leaf and fruit extracts (100 mg/day oleuropein and 20 mg/day hydroxytyrosol) significantly decreased FBG by 4.8% over 2 months (35), while Olive leaf polyphenols (51.1 mg oleuropein and 9.7 mg hydroxytyrosol per day) for 3 months enhanced insulin sensitivity and *β*-cell function in overweight men at risk of metabolic syndrome (19). Another study showed that 500 mg of Olive leaf extract daily for 14 weeks significantly lowered HbA1c and fasting plasma insulin levels in adults with T2DM (36). A recent meta-analysis by Schwingshackl et al., involving four cohort studies (totaling 183,370 participants) and 29 randomized controlled trials (totaling 3,698 participants), confirmed that Olive oil consumption helps prevent and manage T2DM (37). Furthermore, a long-term observational study involving 145,087 US women health professionals suggested a lower risk of T2DM with higher Olive oil intake (38). The antihyperglycemic and antihyperlipidemic effects of Olive plant products are further supported by extensive animal model research (6). Overall, there is substantial pharmacological evidence supporting Olive extracts as a promising natural intervention for the prevention and management of T2DM and related metabolic conditions.

Despite the promising findings, it is important to acknowledge the study’s limitations, which can be addressed in future research. These include the open-label design, lack of randomization and control group, predominantly Caucasian male participants, and a relatively small sample size. Furthermore, there was no systematic measurement of HbA1c at baseline or follow-up. This is because, under the Belgian social healthcare system, HbA1c testing is neither recognized nor reimbursed for non-diabetic or prediabetic individuals, and real-life follow-up of normoglycemic or pre-diabetic patients typically excludes such measurements. A single blood glucose measurement at the start and end of follow-up may also not fully rule out the regression to the mean, particularly for participants with elevated glucose values at baseline. Additionally, body weight data were not available, which could have influenced the metabolic outcomes. While potential confounding factors were not controlled for, the real-life setting and broad inclusion criteria of the study enhance its generalizability. The observed metabolic improvements appear to be genuine, not simply a result of study inclusion or selection bias, underscoring the potential therapeutic effects of the supplemental combination. Future well-designed studies should aim to validate these findings in larger, randomized, blinded, controlled trials with longer follow-up periods. These studies should also incorporate more comprehensive measures such as HbA1c, FBG, PPBG, or, ideally, continuous glucose monitoring with subcutaneous sensors as primary outcomes. Further research should also explore underlying mechanisms in more detail and examine the impact of different dosages on clinical outcomes.

## 6 Conclusion

In conclusion, this retrospective cross sectional observational study, conducted under real-life clinical settings, provides additional clinical evidence, supporting the potential of Amla and Olive fruit extracts as a complementary approach to improving glycemic control in hypercholesterolemic individuals, particularly those with prediabetes or T2DM. The favorable safety and tolerability profile of the supplement underscores its potential for integration into therapeutic regimens. By addressing both glucose and lipid metabolism, this dual-action strategy could enhance overall metabolic health. However, further research is needed to validate these findings, explore the long-term effects, and assess the impact of different dosages on clinical outcomes across diverse populations.

## Data Availability

The original contributions presented in this study are included in the article. Further inquiries can be directed to the corresponding author.
